# Whole‐genome sequencing analysis of Japanese autism spectrum disorder trios

**DOI:** 10.1111/pcn.13767

**Published:** 2024-11-28

**Authors:** Sawako Furukawa, Itaru Kushima, Hidekazu Kato, Hiroki Kimura, Yoshihiro Nawa, Branko Aleksic, Masahiro Banno, Maeri Yamamoto, Mariko Uematsu, Yukako Nagasaki, Tomoo Ogi, Norio Ozaki, Masashi Ikeda

**Affiliations:** ^1^ Department of Psychiatry Nagoya University Graduate School of Medicine Nagoya Japan; ^2^ Medical Genomics Center Nagoya University Hospital Nagoya Japan; ^3^ Department of Psychiatry for Parents and Children Nagoya University Hospital Nagoya Japan; ^4^ Department of Psychiatry Seichiryo Hospital Nagoya Japan; ^5^ Department of Genetics, Research Institute of Environmental Medicine (RIeM) Nagoya University Nagoya Japan; ^6^ Pathophysiology of Mental Disorders Nagoya University Graduate School of Medicine

**Keywords:** autism spectrum disorder, genetics, intellectual disability, phenotype, whole‐genome sequencing

## Abstract

**Aim:**

Autism spectrum disorder (ASD) is a genetically and phenotypically heterogeneous neurodevelopmental disorder with a strong genetic basis. Conducting the first comprehensive whole‐genome sequencing (WGS) analysis of Japanese ASD trios, this study aimed to elucidate the clinical significance of pathogenic variants and enhance the understanding of ASD pathogenesis.

**Methods:**

WGS was performed on 57 Japanese patients with ASD and their parents, investigating variants ranging from single‐nucleotide variants to structural variants (SVs), short tandem repeats (STRs), mitochondrial variants, and polygenic risk score (PRS).

**Results:**

Potentially pathogenic variants that could explain observed phenotypes were identified in 18 patients (31.6%) overall and in 10 of 23 patients (43.5%) with comorbid intellectual developmental disorder (IDD). De novo variants in *PTEN*, *CHD7*, and *HNRNPH2* were identified in patients referred for genetic counseling who exhibited previously reported phenotypes, including one patient with ASD who had profound IDD and macrocephaly with *PTEN* L320S. Analysis of the AlphaFold3 protein structure indicated potential inhibition of intramolecular interactions within PTEN. SV analysis identified deletions in *ARHGAP11B* and *TMLHE.* A pathogenic de novo mitochondrial variant was identified in a patient with ASD who had a history of encephalitis and cognitive decline. GO enrichment analysis of genes with nonsense variants and missense variants (Missense badness, PolyPhen‐2, and Constraint >1) showed associations with regulation of growth and ATP‐dependent chromatin remodeler activity. No reportable results were obtained in the analysis of STR and PRS.

**Conclusion:**

Characterizing the comprehensive genetic architecture and phenotypes of ASD is a fundamental step towards unraveling its complex biology.

Autism spectrum disorder (ASD) is a neurodevelopmental disorder characterized by deficits in social interaction and repetitive behaviors that emerge in early childhood. ASD exhibits significant genetic and phenotypic heterogeneity.^1^, ^2^ Intellectual developmental disorder (IDD) is a major comorbidity that affects 37% of patients of ASD.^3^ Evidence from studies of rare variants with large effect sizes suggests that ASD and IDD exist on a continuum, with genetic overlaps.^4^

Genetic factors play a strong role in ASD, with heritability estimated at approximately 80%.^5^, ^6^ Large‐scale genomic analyses have identified rare single‐nucleotide variants (SNVs)^7^, ^8^, ^9^, ^10^ or rare copy number variations (CNVs)^11^, ^12^, ^13^, ^14^, ^15^ as significant contributors to the ASD pathogenesis. In particular, de novo variants play a crucial role in this process.^13^, ^16^, ^17^ We have identified pathogenic CNVs in ASD and other psychiatric disorders using array comparative genomic hybridization (aCGH) and reported detailed phenotypes of these patients.^11^, ^12^, ^18^, ^19^, ^20^, ^21^, ^22^, ^23^, ^24^, ^25^, ^26^, ^27^ Whole‐exome sequencing (WES) studies have identified ASD risk genes (predominantly expressed in early excitatory and inhibitory neurons) that affect synapses.^7^, ^8^

Both aCGH and WES are affected by detection limitations. In aCGH, the detection accuracy decreases for CNVs <10 kb and for CNVs located in segmental duplications.^12^ aCGH struggles to detect balanced structural variants and accurately identify the break points of CNVs. WES can only examine SNVs and insertion/deletions (INDELs) within the coding region. Whole‐genome sequencing (WGS) allows for more comprehensive genomic analyses and the examination of various types of genetic variants, including small CNVs, other structural variants (SVs), and mitochondrial genome variants.^28^ Furthermore, WGS can identify the break points of SVs at single nucleotide resolution. Certain short tandem repeats (STRs) are associated with neurologic diseases,^29^ including fragile X syndrome, which is associated with *FMR1* and often co‐occurs with ASD. Recently, STRs in *DMPK* and *FXN* have also been linked to ASD.^30^ Although mitochondrial dysfunction has been described as playing a role in ASD pathogenesis,^31^, ^32^, ^33^ the association with ASD remains unclear due to heteroplasmy. Genetic analyses of ASD have primarily focused on rare variants, including de novo variants. However, a contribution by common variants, such as polygenic risk score (PRS), has also been suggested.^34^ WGS enabled us to assess both rare and common variants. Trost et al.^28^ performed the largest WGS study to date of patients with ASD (*N* = 11,312) and reported that rare and dominant variants play a prominent role. The latest review stated that the genetic diagnostic yield of ASD ranges from 30% to 50% as determined by WGS.^35^ Although extensive WGS studies have been conducted in Western countries^28^, ^36^ (*N* = 11,312 and 5205, respectively), such studies have not yet been performed in the Japanese population.

In the current study, we conducted the first WGS analysis of Japanese ASD trios and identified rare pathogenic variants in 57 patients with ASD in whom no pathogenic CNVs were identified through aCGH. We also performed comprehensive genomic studies focusing on rare variants, including SNVs, INDELs, SVs, STRs, mitochondrial variants, and the PRS. By integrating genomic and phenotypic data, we sought to elucidate the clinical significance of pathogenic variants in patients with ASD and thereby enhance the current understanding of the pathogenesis of ASD.

## Methods

### Sample information

The study included 57 patients with ASD (mean ± SD age, 19 ± 10.6 years) and their parents (Table 1). All patients met the criteria for ASD in the *DSM‐5*.^37^ We retrospectively confirmed no ASD diagnosis for all parents. All patients were of Japanese ancestry and recruited from Nagoya University and its affiliated hospitals. Among 57 patients, the proportion of males was 77% (*n* = 44), consistent with the previously reported male to female ratio of 3:1 to 4:1 for ASD. Forty percent of patients (*n* = 23) had IDD, whereas 14% (*n* = 8) were affected by seizures/epilepsy. Other comorbid psychiatric diagnoses included schizophrenia (*n* = 1), attention‐deficit/hyperactivity disorder (*n* = 7), and tic disorder (including Tourette disorder) (*n* = 5). Blood samples were taken from all patients, and blood or saliva was collected from parents. All patients underwent aCGH, which confirmed the absence of pathogenic CNVs.^11^, ^12^ Phenotype data were collected retrospectively from medical records. Written informed consent was obtained from all participants. The ethics committees of the Nagoya University Graduate School of Medicine and associated institutes/hospitals approved this study. The study protocol adhered to the Declaration of Helsinki guidelines.

**Table 1:** Patient information

	Average (range)
Age (years)	19 (6–39)

	Number of patients
Total number of patients	57
Sex	
Male	44
Female	13
Ratio of males (%)	77
Intellectual developmental disorder	23
Profound	5
Severe	4
Moderate	2
Mild	12
Comorbidities	
Seizures/epilepsy	8
ADHD	7
Tic disorder (including Tourette disorder)	5
Syndromic^†^	11
Perinatal asphyxia	4
Neonatal jaundice	4

^†^ Syndromic: patients with complicated medical problems or physical features.

ADHD, attention‐deficit/hyperactivity disorder.

### WGS of ASD trios

Genomic DNA was extracted from whole peripheral blood or saliva, and an MGIEasy FS DNA Prep kit (MGI, Shenzhen, China) was used for library construction. Sequencing was performed using the MGI DNBSEQ‐T7 platform in 150‐bp paired‐end mode. The Genome Analysis Toolkit (GATK) Best Practices was used to detect SNVs/INDELs aligned with the Human Reference Genome hg38 (details are provided in Supplementary Information S1). We excluded variants that failed Variant Quality Score Recalibration, multiallelic sites, and calls with a depth <10. The participants' sex and parent–child relationships, consanguineous marriages, and blood relationships between different trios were examined using PLINK 1.9.^38^ Ancestry was confirmed using PLINK with Phase 3 1000G data. PLINK files from our samples and 1000G data were generated from variant call format (VCF) files, pruned using –geno 0.1 –hwe 0.001 –indep 50 5 2 –maf 0.01 –mind 0.05, and multidimensional scaling analysis was performed to confirm the population (Fig. S1).

### Analysis of SNVs and INDELs

VCF files for SNVs/INDELs were annotated using ANNOVAR^39^ and VEP.^40^ SpliceAI^41^ was used to detect splicing variants with a delta score >0.5. De novo variants were detected using GATK's PossibleDeNovo and Triodenovo v0.06.^42^ Mutual calls of hiConfDeNovo in PossibleDeNovo and Triodenovo (−‐min DQ 7) were extracted. Based on allele frequencies from the Genome Aggregation Database (gnomAD; v4.0.0) for East Asian populations and the Tohoku Medical Megabank Organization, variants with a minor allele frequency (MAF) < 0.001 and AC = 1 were retained. Heterozygous SNVs required GQ ≥99 and 0.3 ≤ AF < 0.8. For heterozygous INDELs, GQ ≥ 90 and 0.3 ≤ AF <0.8 were required. For homozygous SNVs/INDELs, GQ ≥25 and AF ≥0.8 were required. Inherited variants with MAF <0.01 were retained. We performed bam confirmation for potentially pathogenic variants (PPVs) (described below). Additionally, PPVs were validated by Sanger sequencing when the variants were found in patients referred to genetic counseling who exhibited previously reported phenotypes.

### Analysis of SVs

For CNVs > 1 kb, we used CNVkit.^43^ The log2 ratio thresholds were set at −0.6 (deletions) and 0.4 (duplications). For SVs (deletions, duplications, inversions, translocations), we used Manta^44^ and DELLY.^45^ To avoid picking false‐positive variants, we chose “PASS” variants from Manta and “PASS” and “PRECISE” variants from DELLY and then extracted the mutual calls.^28^ To determine the SV count, we integrated the results from CNVkit and mutual calls from Manta and DELLY. We used AnnotSV, version 3.3.4, for SV annotation.^46^ Only rare SVs were selected (MAF <0.01 and singletons). We adopted SVs in which the child was homozygous and both parents were heterozygous, considering those with MAF < 0.2. For STRs, we used ExpansionHunterDenovo (EHdn)^47^ with outlier analysis and included *z* > 10. For known STRs, we used ExpansionHunter^48^ and visualized them using REViewer.^49^ De novo variants and PPVs were performed using bam confirmation.

### Analysis of mitochondrial variants

We detected mitochondrial variants using the GATK mitochondrial pipeline. Filtered VCF files were annotated using ANNOVAR^39^ and mitimpact3D.^50^, ^51^

### Prioritization of potentially pathogenic variants

PPVs were defined as follows. We chose ASD/IDD genes from the SFARI Gene database,^52^ DECIPHER,^53^ ClinGen Intellectual Disability and Autism Gene Curation Expert Panel,^54^ and gene panels from the Genomics England PanelApp. We included known disease genes associated with neurodevelopmental phenotypes, including brain abnormalities, and epilepsy. Additionally, synapse genes were identified using SynGO.^55^ For de novo SNVs/INDELs, we included variants of ASD/IDD genes. For homozygous (both parents are heterozygous) and maternally inherited hemizygous variants, we included protein‐truncating variants (PTVs) or missense variants with Missense badness, PolyPhen‐2, and Constraint (MPC) >2 in ASD/IDD genes. Compound heterozygous variants were considered if one variant was a PTV or a missense variant with MPC >2 or if both variants were PTVs or missense variants with MPC >1 in ASD/IDD genes. For SVs, we adopted variants overlapping exonic regions of ASD/IDD genes when variants were de novo, homozygous (both parents are heterozygous), or maternally inherited hemizygous. For mitochondrial variants, we adopted known pathogenic variants annotated by ClinVar with >5% heteroplasmy.

### Gene Ontology enrichment analysis

Gene Ontology (GO) enrichment analysis was performed using Metascape.^56^ We input 66 genes harboring PTVs and missense variants with MPC >1 from de novo, homozygous (both parents are heterozygous), maternally inherited hemizygous, and compound heterozygous SNVs/INDELs (Table S1). Results at *P* < 0.01 were visualized using a network plot (Fig. S2).

### Protein structure assessment

AlphaFold3^57^, ^58^ was used to predict and compare protein structures with and without potentially pathogenic missense variants. We prepared fasta files of the amino acid sequences for both the variant‐derived protein and wild‐type protein. We predicted the three‐dimensional structures of the proteins using AlphaFold3 (https://golgi.sandbox.google.com), and the predicted structures were visualized using PyMOL. For heterozygous variants, we designed fasta files based on the allele with the variant for the prediction of the protein's tertiary structure.

### Noncoding region analysis

Limited clinical interpretation is currently available for noncoding variants, but cis‐regulatory elements (CREs) of genes associated with the disease of interest within target tissues may induce disease development.^59^, ^60^ Therefore, given that ASD risk genes are highly expressed and converge during fetal brain development,^7^, ^61^ we employed fetal brain‐specific CREs (b‐CREs) identified using PsychSCREEN by PsyENCODE.^62^ We evaluated de novo variants that overlapped with the fetal b‐CREs in ASD/IDD genes.

### PRS calculation

To assess the impact of common variants, we calculated the PRS from iPSYCH‐PGC ASD genome‐wide association study (GWAS) results.^34^ Multiallelic sites and duplicated variants were excluded from PLINK files generated from VCFs, and only SNPs were included. QC was performed using –geno 0.1 –hwe 1e‐6 –maf 0.01 –mind 0.1. We also generated PLINK files from 1000G JPT and extracted mutual variants for our samples and then merged them with our samples. Next, we calculated the PRS using PRS‐csx^63^ to perform cross‐population polygenic predictions, as the base data were generated from the European population. PRSs were compared between groups using two‐sample independent *t* tests.

## Results

### WGS of the Japanese ASD cohort

**Fig. 1:**
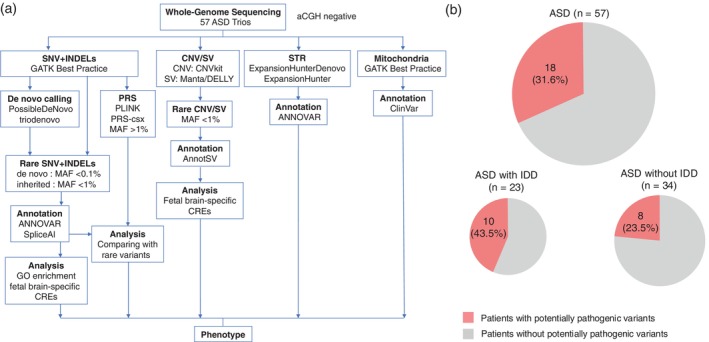
Overview of our study. (a) Workflow of our study. We analyzed 57 autism spectrum disorder (ASD) trios in whom no pathogenic copy number variations (CNVs) were identified by array comparative genomic hybridization (aCGH). We conducted a whole‐genome sequencing (WGS) analysis using a comprehensive pipeline targeting various types of variants. (b) Proportion of patients with potentially pathogenic variants. The proportion is shown among all patients with ASD and among patients with ASD with/without intellectual developmental disorder (IDD). CRE, cis‐regulatory element; GO, Gene Ontology; INDEL, insertion/deletion; MAF, minor allele frequency; PRS, polygenic risk score; SNV, single‐nucleotide variant; STR, short tandem repeat; SV, structural variant.

We performed WGS analysis of 57 Japanese ASD trios and detected rare SNVs/INDELs, SVs (including CNVs), STRs, and mitochondrial variants and determined the PRS (Fig. 1a). The average read depth was 27.4×. After QC and filtering, an average of 3,769,764 variants were identified per individual, of which 3,433,920 (91.1%) were SNVs and 335,844 (8.9%) were INDELs. An average of 65,141 rare variants (MAF <0.01) were detected per genome, of which 63,865 (98.0%) were heterozygous and 1276 (2.0%) were homozygous. We identified 4107 de novo SNVs/INDELs among all probands, with an average of 72 de novo SNVs/INDELs (SD: 18.7). Among these SNVs/INDELs, 51 were in the coding region, including four PTVs, 33 missense variants, and 14 synonymous SNVs. Only one intronic de novo variant potentially affecting splicing (delta score 0.75) was identified (Table 2A). An average of 67.86 rare SVs were detected per proband. A total of 3.19 rare de novo SVs were detected per proband (Table 2B). In addition, we detected one de novo mitochondrial variant.

**Table 2:** Summary of rare variant counts among Japanese patients with ASD.

(A) Summary of rare SNVs/INDELs	
Mean number of SNVs/INDELs (per individual)	3,769,764
SNVs	3,433,920
INDELs	335,844
Mean number of rare SNVs/INDELs (per individual)	65,141
Heterozygous	63,865
Homozygous	1276
Mean number of rare de novo SNVs/INDELs (per individual)	72.05
Total number of rare de novo SNVs/INDELs	4107
Coding variants	51
Noncoding variants in gene	1946
Intergenic variants	2110
Classification of coding rare de novo SNVs/INDELs	
Stopgain	2
Frameshift insertion	1
Frameshift deletion	1
Missense	33
Synonymous SNVs	14
De novo splicing SNVs/INDELs	1

(B) Summary of rare SVs (including CNVs)	
Total number of rare SVs	3868
DEL	2525
DUP	982
INS	87
INV	14
TRA	260
Mean number of rare SVs (per individual)	67.86
Mean length of rare SVs (kb)	73.19
Median length of rare SVs (kb)	6.98
Total number of rare de novo SVs	182
Mean number of rare de novo SVs (per individual)	3.19

ASD, autism spectrum disorder; CNV, copy number variation; DEL, deletion; DUP, duplication; INDEL, insertion/deletion; INS, insertion; INV, inversion; SNV, single‐nucleotide variant; SV, structural variant; TRA, translocation.

### Potentially pathogenic variants identified in the Japanese ASD cohort

After filtering, PPVs were prioritized among ASD/IDD genes, and the inheritance patterns and impact on protein function were assessed in silico. A total of 23 PPVs were identified in 18 of the 57 patients (31.6%) (Fig. 1b and Table S2). Of these 23 variants, 19 (82.6%) were SNVs/INDELs, two (8.7%) were SVs, and two (8.7%) were mitochondrial variants.

Among the 19 potentially pathogenic SNVs/INDELs, 12 were de novo SNVs/INDELs in ASD/IDD genes (Table 3A): *PTEN*, *CHD7*, *HNRNPH2*, *BSN*, *HNRNPR*, *MKX*, *SMARCA4*, *RPS6KA3*, *GLUL*, *TRIP12*, *TEK*, and *PCLO*. The PTVs in *CHD7* and *BSN* were novel. We detected compound heterozygous PPVs in *HLCS*, *EPPK1*, *MADD*, *SLC25A10*, *CHD5*, and *JARID2* (Table 3A). Maternally inherited hemizygous PPVs were found in *GLRA2* (Table 3A).

**Table 3:** Potentially pathogenic variants in the ASD cohort.

(A) Potentially pathogenic SNVs and INDELs						
Sample	Position (hg38)	Variant	Inheritance	pLI	SFARI	IDD
**Patient 1** ^†^	**chr10:g.87961051T>C**	***PTEN* p.L320S**	**De novo**	**0.048**	**1S**	**Profound**
**Patient 2** ^†^	**chr8:g.60852995G>A**	***CHD7* p.W2090X**	**De novo**	**1**	**1S**	**Mild**
**Patient 3** ^†^	**chrX:g.101412410T>A**	***HNRNPH2* p.M141K**	**De novo**		**1**	**Severe**
Patient 4	chr3:g.49654526‐>C	*BSN* p.R1659Qfs*23	De novo	1		Moderate
Patient 6	chr1:g.23318521C>T	*HNRNPR* p.D327N	De novo	1	2S	
Patient 7	chr10:g.27741428G>A	*MKX* p.L89F	De novo	0.782	1	
Patient 8	chr19:g.11041349C>T	*SMARCA4* p.R1405W	De novo	1	1	
Patient 9	chrX:g.20165002G>A	*RPS6KA3* p.P554L	De novo		2S	Borderline
Patient 10	chr1:g.182386318G>A	*GLUL* p.T138I	De novo	0.999		
Patient 11	chr2:g.229802327G>A	*TRIP12* p.T1044I	*De novo*	1	1S	
Patient 12	chr9:g.27183504T>C	*TEK* p.I359T	De novo	1	1	Mild
Patient 13	chr7:g.82914875T>C	*PCLO* p.M4371V	De novo	1	2	Mild
Patient 14	chr21:g.36759828C>T	*HLCS* p.R712Q	Maternal	1.26E‐11		Mild
	chr21:g.36966576GA>G	*HLCS* p.L21Pfs*32	Paternal			
Patient 1	chr8:g.143873145T > TG	*EPPK1* p.R37Qfs*80	Maternal	0.123	2	Profound
	chr8:g.143872401C > T	*EPPK1* p.G285S	Paternal			
Patient 15	chr11:g.47289788G > A	*MADD* c.2553‐79G > A	Maternal	0		Moderate
	chr11:g.47289022G > A	*MADD* p.G903D	Paternal			
Patient 15	chr17:g.81720046G > A	*SLC25A10* p.R278H	Maternal	2.42E‐10		
	chr17:g.81715055G > A	*SLC25A10* p.A66T	Paternal			
Patient 6	chr1:g.6142258T > A	*CHD5* p.E769V	Maternal	1		
	chr1:g.6154701T > C	*CHD5* p.Q235R	Paternal			
Patient 16	chr6:g.15487405C > T	*JARID2* p.R257W	Maternal	1	2	
	chr6:g.15517216T > C	*JARID2* p.V1169A	Paternal			
Patient 17	chrX:g.14690789C > T	*GLRA2* p.A337V	Maternal		2	Mild

(B) Potentially pathogenic SVs							
Sample	Position (CytoBand) (hg38)	SV type	ASD/IDD gene	Inheritance (zygosity)	Length (kb)	SFARI	IDD
Patient 14	chr15:30587397–30799740 (15q13.2)	DEL	*ARHGAP11B*	Both parents (homozygous)	212	2	Mild
Patient 18	chrX:155543337–155555264 (Xq28)	DEL	*TMLHE*	Maternal (hemizygous)	11.9	2	

(C) Potentially pathogenic mitochondrial variants						
Sample	Variant	Gene	Inheritance	Heteroplasmy/homoplasmy	ClinVar	IDD
Patient 5	m.5540G > A	*MT‐TW*	*De novo*	6.7% (heteroplasmy)	Pathogenic	Mild
Patient 1	m.14674T > C	*MT‐TE*	Maternal	Homoplasmy (mother: homoplasmy)	Likely pathogenic	Profound

^†^ Patients presenting with previously reported phenotypes and who were referred to genetic counseling are shown in bold. ASD, autism spectrum disorder; IDD, intellectual developmental disorder; INDEL, insertion/deletion; SNV, single‐nucleotide variant; SV, structural variant.

With regard to SVs, we identified two potentially pathogenic rare CNVs (Table 3B), one of which was a homozygous deletion spanning 212 kb in the 15q13.2 region, which includes the human‐specific gene *ARHGAP11B*.^64^ The other potentially pathogenic rare CNV was a hemizygous deletion of 12 kb (including exon 2) of *TMLHE*, an ASD‐associated gene^65^, ^66^ involved in carnitine metabolism.^67^ We did not detect any de novo SVs that included coding regions of ASD/IDD genes. In addition, inherited variants were detected in ASD/IDD genes (Table S3). One of these inherited variants was a duplication of *RHEB*, maternally derived, in a patient with ASD who had a brain tumor. RHEB is a member of the mechanistic target of rapamycin (mTOR) pathway and regulates mTORC1, which is involved in cell growth and proliferation.^68^ The other inherited variant was a paternal duplication of the 17p11.2 region, including *NF1*. Compound heterozygous events involving SNVs/INDELs and SVs were identified on *CNTNAP3B* (a maternal frameshift deletion and paternal large deletion); however, the clinical significance of these events remains unclear.

We also investigated STRs from our ASD cohort. Outlier motifs of EHdn results did not include 10 known ASD‐associated STRs (*DMPK*, *FGF14*, *CACNB1*, *FXN*, *CDON*, *MYOCD*, *MBOAT7*, *IL1RAPL1*, *FMR1*, and *IGF1* ^30^) or any STRs located within ASD/IDD genes. We also confirmed that no known pathogenic STRs for other diseases were present.

For mitochondrial variants, we evaluated those with >5% heteroplasmy in ASD probands. Two known pathogenic mitochondrial variants were identified, one which constituted 6.7% of the load of the de novo variant in *MT‐TW* (m.5540G>A) associated with mitochondrial encephalopathy, lactic acidosis, and stroke‐like episodes (MELAS), and the other a maternally inherited homoplasmy in *MT‐TE* (m.14674T >C) associated with primary mitochondrial disease (Table 3C).

### GO enrichment analysis

**Fig. 2:**
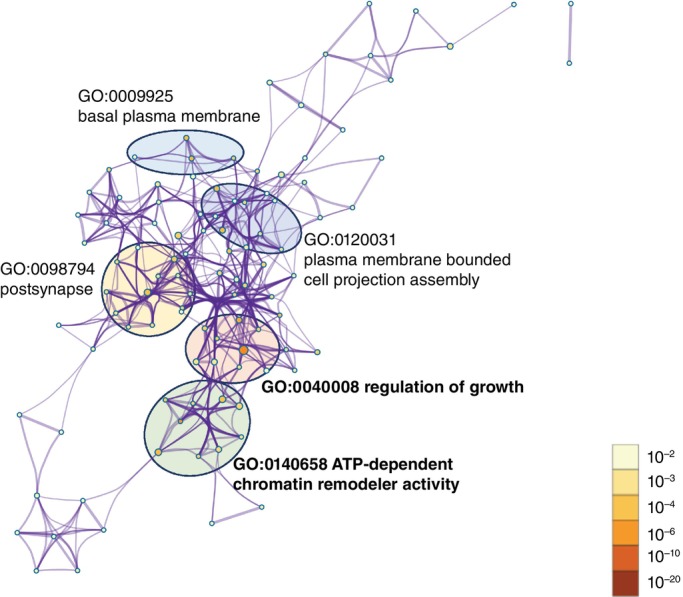
Gene Ontology (GO) enrichment analysis. Network plot showing the top five statistically significant GO terms (*P* < 0.01) drawn using Metascape. Enriched GO terms shown in bold were GO:0040008: regulation of growth (*P* = 9.086E‐08, *q* = 0.00199) and GO:0140658: ATP‐dependent chromatin remodeler activity (*P* = 1.149E‐06, *q* = 0.0126). Three additional terms included GO:0009925: basal plasma membrane (*p* = 3.80E‐5, *q* = 0.149), GO:0120031: plasma membrane bound cell projection assembly (*p* = 4.25E‐05, *q* = 0.149), and GO:0098794: postsynapse (*p* = 7.68E‐05, *q* = 0.170).

GO enrichment analysis was performed using Metascape.^56^ We input 66 genes harboring PTVs and missense variants with MPC >1 from de novo, homozygous (both parents were heterozygous), maternally inherited hemizygous, and compound heterozygous SNVs/INDELs (Table S1). Enriched GO terms included GO:0040008: regulation of growth (*P* = 9.09E‐08, *q* = 0.00199) and GO:0140658: ATP‐dependent chromatin remodeler activity (*P* = 1.15E‐06, *q* = 0.0126) (Fig. 2). Among 11 genes in the most‐enriched term, regulation of growth (*ERBB4*, *JARID2*, *PTEN*, *RPS6KA3*, *SMARCA4*, *LTBP4*, *HDAC6*, *SEMA6B*, *CHD7*, *EPPK1*, *EGLN2*), eight (72.7%) were ASD/IDD genes.

### Evaluating the impact of variants on protein structure using AlphaFold3

**Fig. 3:**
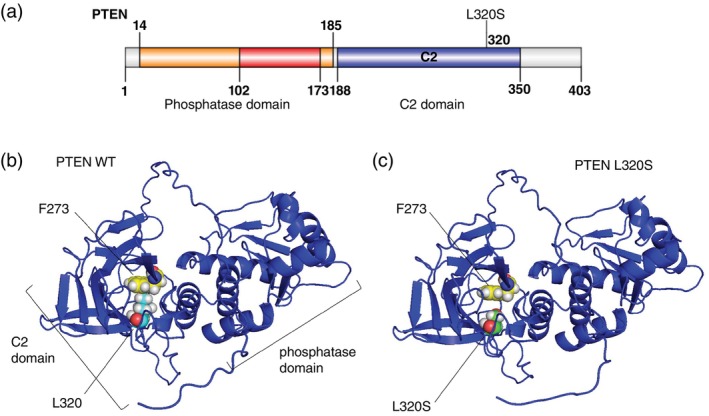
PTEN L320S inhibits intramolecular interactions. (a) The domain structure of PTEN (403 amino acids). Figure shows the major functional domains determined using InterPro (https://www.ebi.ac.uk/interpro) and visualized using IBS.^104^ L320S lies in the C2 domain. (b, c) Tertiary structure of PTEN predicted using AlphaFold3 (B: wild type [WT], C: L320S). L320/L320S and F273 are shown as spheres.

We utilized AlphaFold3, a state‐of‐the‐art AI tool,^57^, ^58^ to investigate whether PPVs in our patients with ASD alter the tertiary structure of proteins or affect intramolecular interactions. We investigated 13 potentially pathogenic missense variants (Table 3). Analysis of a de novo missense variant in *PTEN* (NM_000314:c.959T>C:p.[Leu320Ser]) in a patient with ASD, profound IDD, and macrocephaly revealed an increased distance between residues PTEN L320S and F273 in the tertiary structure of L320S compared with the wild type (Fig. 3a–c). The predictive accuracy for this region was high (pLDDT >90). Previous experimental studies showed that L320S blocks PTEN localization at the plasma membrane and nucleus without suppressing enzymatic activity or affecting lipid phosphatase activity.^69^ Crystal structure analyses of PTEN indicated that L320 and F273 interact^70^ to maintain plasma membrane and nuclear localization.^69^ Our results were consistent with previous findings, demonstrating the crucial role of PTEN L320S in the function of PTEN. Although the recently developed AlphaFold3 tool is excellent for predicting biomolecular interactions,^57^ no clear examples of variants located within the binding sites were found using AlphaFold3.

### Analysis of noncoding regions

Given that ASD risk genes are highly expressed and converge during fetal brain development,^7^, ^61^ we focused on fetal b‐CREs^71^ in ASD/IDD genes. For SNVs/INDELs, we found eight de novo SNVs that overlapped with fetal b‐CREs in ASD/IDD genes in eight patients. The affected genes were *FOXP2*, *DMWD*, *EXT2*, *DPP10*, *CNTN3*, *RAB2A*, *NTRK2*, and *MOCS1*. Among the eight patients, six did not have any PPVs in coding regions. We did not identify any de novo SVs that overlapped with fetal b‐CREs in ASD/IDD genes.

### Clinical phenotypes of patients with potentially pathogenic variants

As previously indicated, PPVs were identified in 18 of 57 patients (31.6%). Among the 23 patients who had comorbid IDD (Table 1), PPVs were detected in 10 (43.5%) (Fig. 1b). Among the 34 patients without IDD (Table 1), PPVs were detected in eight (23.5%) (Fig. 1b). Although not statistically significant (*P* = 0.245), patients with comorbid IDD exhibited a 20% higher prevalence of PPVs than patients without IDD. Detailed clinical information for the 18 patients with PPVs is provided in Table 4 and S2.

**Table 4:** Clinical data for five notable patients with potentially pathogenic variants

	Patient 1	Patient 2	Patient 3	Patient 4	Patient 5
Diagnosis (*DSM‐5*)	ASD, profound IDD (FSIQ <20)	ASD, mild IDD (FSIQ 62)	ASD, severe IDD, epilepsy	ASD, moderate IDD (FSIQ 42), epilepsy	ASD, mild IDD (FSIQ 54), epilepsy
Sex, age (years)	Male, 17	Female, 25	Male, 38	Male, 14	Male, 13
Potentially pathogenic variants	*PTEN* L320S (*de novo*)	*CHD7* W2090X (de novo)	*HNRNPH2* M141K (de novo)	*BSN* R1659Qfs*23 (de novo)	*MT‐TW* m.5540G>A (de novo)
	*EPPK1* R37Qfs*80 (compound heterozygous)				
	*EPPK1* G285S (compound heterozygous)				
	*MT‐TE* m.14674T > C (maternally inherited)				
Family history	Paternal first cousin: ASD Paternal aunt: IDD	Not remarkable	Sister: schizophrenia	Mother: depressive state	Not remarkable
Birth	Preterm infant (32w0d, 1940g) No asphyxia	Feeding difficulties Hearing impairment	NA	NA	Not remarkable
Dysmorphic features	Macrocephaly	Patent ductus arteriosus Deformity of the right external ear	Almond‐shaped eyes Short palpebral fissures Flat philtrum Thin upper lip Micrognathis Triangular face Pointed chin Retroverted ears High‐pitched voice	NA	Not remarkable
Developmental milestones	Head control: 5 months Unsupported sitting: 12 months Independent walking: 23 months No meaningful words observed to date	Delayed head control Lack of coordinated eye movement Generalized hypotonia Difficulty with fine motor skills	NA	Delayed language development	Delayed language development Dysarthria Difficulties with fine motor skills Experienced cognitive decline
Social interactions/communication skills	Understanding of simple, routine verbal instructions	Stubborn Exhibited stranger anxiety but affectionate towards preferred individuals	Sending an excessive number of letters to caregivers and relatives	Unprovoked aggression towards sister and classmates Strongly persisted in imposing his opinions and desires	One‐sided conversations Poor eye contact Feeling fear in new situations
Restricted/repetitive behaviors and interests	Frequently manipulates genitals during restlessness or idleness	Refuses to move from a seat until producing a cough	Being preoccupied with his anus, leading to frequent bathroom visits	Struggles with new situations and avoids activities that do not interest him Refuses to return home from walks until seeing trains	Strong fixation on sugar
Head CT or brain MRI	Not remarkable	MRI: olfactory bulbs, sulci, and nerves not identifiable; hypoplastic semicircular canals and cochlea	MRI: arachnoid cyst	NA	Not remarkable

ASD, autism spectrum disorder; CT, computed tomography; FSIQ, Full‐Scale Intelligence Quotient; IDD, intellectual developmental disorder; MRI, magnetic resonance imaging; NA, not available.

Here, we present five patients of particular interest (Table 4). In these patients, we identified potentially pathogenic de novo SNVs/INDELs (Table 3) in three syndromic ASD genes: *PTEN*, *CHD7*, and *HNRNPH2*. These variants were found in three patients referred to genetic counseling who exhibited previously reported phenotypes. Patient 1 had ASD with profound IDD and macrocephaly and a known de novo variant (NM_000314:c.959T>C:p.[Leu320Ser]) in *PTEN* (macrocephaly/autism syndrome, MIM: 605309).^72^ This patient also had potentially pathogenic compound heterozygous variants in *EPPK1* and a maternally inherited mitochondrial variant (m.14674T>C) (Tables 3 and 4). Patient 2 had ASD with mild IDD, patent ductus arteriosus, hypotonia, and congenital hearing loss and a de novo PTV in *CHD7* (ASD, CHARGE syndrome, MIM: 214800)^73^ (NM_017780:c.6270G>A:p.[(Trp2090*]). Patient 3 had ASD, severe IDD, epilepsy, and dysmorphic features and a de novo *HNRNPH2* variant (NM_019597:c.422T>A:p.(Met141Lys]) (intellectual developmental disorder, X‐linked syndromic, Bain type, MIM: 300986).^74^, ^75^ Before undergoing genetic testing in this study, the patients had not been suspected of having these syndromes. Patient 4 had ASD, moderate IDD, and epilepsy and a novel PTV in *BSN*. Patient 5 had ASD, mild IDD, and epilepsy and a 6.7% load of a known pathogenic de novo mitochondrial variant for MELAS. This patient had a history of encephalitis, which was accompanied by a subsequent cognitive decline. He frequently experienced headaches and nausea.

### PRS determination

We calculated the PRS in our patients and the 1000G (Japanese) sample from a recent ASD GWAS.^34^ There was no difference in PRS between our patients and the 1000G sample (*t* = 0.26, *P* = 0.795) (Fig. S2A). We then compared the PRS for patients with/without PPVs to assess the interactions for PRS and rare variant burden and found no difference (*t* = 0.037, *P* = 0.970) (Fig. S2B). We also compared the mean PRS for patients with/without IDD and found no significant difference (*t* = 0.361, *P* = 0.720) (Fig. S2C). The mean PRS for patients with moderate, severe, and profound IDD was not significantly different compared with that of patients with mild IDD (*t* = 1.78, *P* = 0.090) (Fig. S2D).

## Discussion

We conducted a WGS analysis of 57 ASD trios, investigating variants such as SNVs, SVs, STRs, and mitochondrial variants, and determination of the PRS (Fig. 1a). PPVs were detected in 18 of 57 patients (31.6%). Our detection rate of PPVs was consistent with rates reported in a previous review article, which indicated genetic diagnostic yields of ASD of 30% to 50% as determined by WGS.^35^ Also, in line with previous studies,^76^ we observed a higher prevalence of PPVs in patients with ASD who have IDD than in patients without IDD (43.5% vs 23.5%), although the difference was not statistically significant (Fig. 1b). Among PPVs, 82.6% were SNVs/INDELs, 8.7% were SVs, and 8.7% were mitochondrial variants. The largest previous studies showed that the genomic architecture of ASD‐associated variants consisted of 52% SNVs/INDELs, 46% SVs, and 2% mitochondrial variants.^28^ The high proportion of SNVs/INDELs among our PPVs can be attributed to the exclusive inclusion of patients in whom no pathogenic variants were identified through aCGH.

Four patients with ASD had multiple PPVs, all presenting with IDD and/or epilepsy. The accumulation of rare pathogenic variants may have contributed to the more severe phenotypes of these patients.^77^ We also identified a patient with ASD who had profound IDD and macrocephaly (patient 1) and harbored three PPVs: *PTEN* L320S, compound heterozygous variants in *EPPK1*, and a mitochondrial variant (m.14674T>C) (Tables 3 and 4). AlphaFold3 analysis revealed an increased distance between residues PTEN L320S and F273 in the tertiary structure of L320S compared with the wild type (Fig. 3b,c), consistent with previous findings. Although a Japanese patient in a previous study who had the same de novo L320S variant exhibited milder phenotypes,^72^ patient 1 in our study presented with more severe IDD (nonverbal in early 20s vs two‐word phrases at 30 months). Patient 1 had no perinatal episode, a significant risk factor for IDD, suggesting that these additional variants may have contributed to his severe phenotype. As PTEN interacts with mitochondria^78^ and L320S lies in the C2 domain, which is crucial for membrane binding,^79^ *PTEN* L320S and m.14674T>C may act in concert to contribute to the phenotype in patient 1. Future studies using patient‐derived induced pluripotent stem cells are necessary to investigate the mechanism.^80^

We also detected rare compound heterozygous variants in *JARID2* and *CHD5*, genes typically associated with ASD/IDD through autosomal dominant inheritance. Although variants in these genes are often considered benign when inherited from a parent, our findings suggest that biallelic variants contribute to the phenotype through a recessive inheritance mechanism. This observation highlights the importance of considering recessive inheritance—even in regard to genes typically associated with dominant inheritance—when evaluating variant pathogenicity in patients with ASD/IDD.

We identified a novel PTV of *BSN* in a patient with ASD, moderate IDD, and epilepsy (patient 4). The protein bassoon, encoded by *BSN*, is a presynaptic scaffolding protein essential for regulating neurotransmitter release.^81^, ^82^ Although *BSN* has not been implicated in ASD, an association with epilepsy has been suggested.^83^ Considering that *PCLO*, the gene encoding piccolo, which forms the presynapse along with bassoon, is associated with ASD,^7^, ^84^ the PTV in *BSN* may contribute to the phenotype in the case of patient 4.

We initially aimed to identify pathogenic variants of medium size (50–10,000 bp), particularly de novo variants, which are undetectable by aCGH/WES. Although we did not detect any de novo variants encompassing coding regions of ASD/IDD genes, we did identify de novo SNVs in fetal b‐CREs. CREs regulate gene expression tissue/temporal specifically,^85^, ^86^, ^87^, ^88^ and, therefore, these de novo variants might impact the ASD pathogenesis during the embryonic period. As evidence continues to accumulate, variants of this nature may eventually contribute to diagnostic yield.

GO enrichment analysis of genes with nonsense variants and missense variants (MPC >1) showed associations with regulation of growth and ATP‐dependent chromatin remodeler activity. The most‐enriched term, regulation of growth, is associated with the mTOR signaling pathway, which plays an important role in syndromic ASD/IDD, such as FMR1 in fragile X syndrome, TSC in tuberous sclerosis, MECP2 in Rett syndrome, and PTEN.^89^, ^90^, ^91^, ^92^ Chromatin remodeler activity is also associated with the pathophysiology of ASD,^11^, ^12^, ^92^, ^93^, ^94^ suggesting that there are similarities between the biological characteristics of our patients and those of other ethnic groups.

The protein structural changes we predicted were consistent with those of a previous experimental study.^69^ However, because AlphaFold2 predictions are based on known wild‐type domains,^95^ the accuracy of its predictions in terms of the structural impact of missense variants remains uncertain. Nevertheless, repeated model generation consistently showed an increased inter‐residue distance, thus warranting special attention. Furthermore, the recently developed AlphaFold3 tool,^57^ which exhibits improved accuracy in predicting biomolecular interactions, is expected to accelerate research regarding the interactions between variant‐derived proteins and DNA or RNA.

Mitochondrial dysfunction has been described in the ASD pathogenesis.^31^, ^32^, ^33^ However, due to heteroplasmy, such association studies are more complicated than studies of nuclear genome variants. A previous study revealed that pathogenic heteroplasmies in mitochondrial DNA were more enriched in ASD probands.^96^ In addition, patient 5 of our study had a 6.7% load of de novo heteroplasmy. With regard to MELAS, individuals harboring the best‐characterized variant, m.3243A>G in the *MT‐TL1* gene, have a heteroplasmy load of >50%. This variant is also associated with seizures and ASD.^97^ Another variant, m.3271T>C in the *MT‐TL1* gene, exhibits a 10% heteroplasmy load in patients with MELAS.^98^ As more mitochondrial genome variants are identified and heteroplasmy loads are determined in larger ASD cohorts, the continuity between ASD and known mitochondrial diseases is expected to become more evident.

We calculated the PRS for our cohort based on WGS data. Despite the small sample size, patients with moderate, severe, or profound IDD appeared to have a higher PRS than patients with mild IDD, although the difference was not statistically significant (Fig. S2D). These results suggest that common variants may have an additive effect on phenotype, consistent with previous studies.^99^, ^100^, ^101^ As WGS becomes mainstream and sample sizes increase, our understanding of the impact of rare and common variants will increase.

Our study has several strengths. First, we comprehensively analyzed a wide range of variants using WGS and identified PPVs that could not be detected by aCGH/WES. Second, we used AlphaFold3 to predict protein structural changes, which enabled a more‐detailed evaluation of variant pathogenicity. Third, we calculated the PRS, which provided insights into the interactions between rare and common variants in ASD.

However, our study also has limitations. First, our small sample size (*n* = 57) limits the generalizability of our results. Second, blood‐based sampling may miss somatic variants or epigenetic contributors in the brain.^102^ Third, it is difficult to decipher complex genomic regions (long repeats, centromeric areas) using short‐read WGS.

Among our patients, 11 had IDD of greater than moderate severity, suggesting that the genetic backgrounds of these patients were distinct from their parents. In patients with severe phenotypes, variants in *PTEN*, *CHD7*, and *HNRNPH2* were identified, leading to referral for genetic counseling. However, no explanatory variants were detected in some patients, particularly females with ASD and profound IDD, despite a high likelihood of pathogenic variant discovery.^103^ Variants in these regions may be responsible for the observed phenotypes in these cases. Further analyses focusing on patients with severe phenotypes and variants that cannot be identified using current methods are warranted.

## Disclosure statement

S.F. and I.K. declare no conflicts of interest. N.O. and M.I. are editorial board members of *Psychiatry and Clinical Neurosciences* and coauthors of this article. To minimize bias, they were excluded from all editorial decision‐making related to the acceptance of this article for publication. N.O. has received research support, speakers' honoraria, and/or royalties from or has served as a joint researcher with or consultant to Sumitomo Pharma, Otsuka, KAITEKI, Takeda, Ricoh, Meiji Seika Pharma, Taisho Pharma, Mochida, Shionogi, Mitsubishi Tanabe, Tsumura, EA Pharma, Eli Lilly, Daiichi Sankyo, MSD, Lundbeck Japan, Viatris, Eisai, Mochida, Kyowa Pharmaceutical Industry, Nihon Medi‐Physics, Kyowa Kirin, Janssen pharma, Yoshitomi Pharmaceutical, Nippon Chemiphar, Medical Review, Nippon Boehringer Ingelheim, Ono Pharmaceutical, Woolsey Pharmaceuticals, and SUSMED, outside the submitted work. M.I. has received speakers' honoraria from Sumitomo Pharma, Eisai, Otsuka, Tanabe Mitsubishi, Mochida, Takeda, Meiji Seika Pharma, EA Pharma, Viatris, MSD, Janssen, Lundbeck, and Yoshitomi. All other authors report no biomedical financial interests or potential conflicts of interest.

## Author contributions

S.F., I.K., and N.O. designed the study. S.F. performed experiments and analyzed the data. I.K., Hidekazu K., Hiroki K., M.B., M.Y., Y.N., M.U., and N.O. recruited participants and/or collected DNA samples or phenotype data. S.F. wrote the manuscript draft, I.K. supervised the study, and the others commented on and refined the manuscript. All authors carefully read the paper and approved the final manuscript.

## Supporting information


**Table S1.** Input genes for enrichment analysis.

**Table S2.** Clinical data for all patients with potentially pathogenic variants.

**Table S3.** All SVs including CNVs overlapping exonic regions of ASD/IDD genes.

**Fig. S1.** Ancestry confirmation
Multidimensional scaling (MDS) analysis was performed to exclude population outliers. Principal component analysis using the 1000 Genomes Project reference panel (phase 3) detected no patients with probable ancestries outside the east Asian population and ensured that all probands were of apparent Japanese origin. Red, light green, pink, and yellow circles indicate CEU (Utah residents [CEPH] with Northern and Western European ancestry), CHB (Han Chinese in Bejing, China), JPT_1000G (Japanese in Tokyo, Japan), and YRI (Yoruba in Ibadan, Nigera) in 1000G, respectively. Blue circles indicate our sample.
**Fig. S2.** Polygenic risk score (PRS). (A) PRS distribution of our patients and a sample of Japanese patients from 1000G (JPT_1000G). The mean PRS for our patients and JPT_1000G was 6.57E‐07 and 6.51E‐07, respectively (*t* = 0.26, *P* = 0.795). (B) PRS distribution plotted separately for patients with and without potentially pathogenic variants. The mean PRS for patients with/without potentially pathogenic variants was 6.58E‐07 and 6.56E‐07, respectively (*t* = 0.037, *P* = 0.970). (C) PRS distribution plotted separately for patients with and without intellectual developmental disorder (IDD). The mean PRS for patients with and patients without IDD was 6.64E‐07 and 6.51E‐06, respectively (*t* = 0.361, *P* = 0.720). (D) PRS distribution plotted separately for patients with moderate, severe, and profound IDD; patients with mild IDD; and patients without IDD. The mean PRS for patients with moderate, severe, and profound IDD and those with mild IDD was 7.03E‐07 and 6.28E‐07, respectively (*t* = 1.78, *P* = 0.089).
