# Supplementary material for: Whole‐genome sequencing analysis of Japanese autism spectrum disorder trios
Source: Psychiatry Clin Neurosci. 2024 Nov 28;79(3):87–97. doi: 10.1111/pcn.13767 (PMC11874045; doi:10.1111/pcn.13767)
Supplement: Supplementary file 4 — Fig. S1. Ancestry confirmation Multidimensional scaling (MDS) analysis was performed to exclude population outliers. Principal component analysis using the 1000 Genomes Project reference panel (phase 3) detected no patients with probable ancestries outside the east Asian population and ensured that all probands were of apparent Japanese origin. Red, light green, pink, and yellow circles indicate CEU (Utah residents [CEPH] with Northern and Western European ancestry), CHB (Han Chinese in Bejing, China), JPT_1000G (Japanese in Tokyo, Japan), and YRI (Yoruba in Ibadan, Nigera) in 1000G, respectively. Blue circles indicate our sample. Fig. S2. Polygenic risk score (PRS). (A) PRS distribution of our patients and a sample of Japanese patients from 1000G (JPT_1000G). The mean PRS for our patients and JPT_1000G was 6.57E‐07 and 6.51E‐07, respectively (t = 0.26, P = 0.795). (B) PRS distribution plotted separately for patients with and without potentially pathogenic variants. The mean PRS for patients with/without potentially pathogenic variants was 6.58E‐07 and 6.56E‐07, respectively (t = 0.037, P = 0.970). (C) PRS distribution plotted separately for patients with and without intellectual developmental disorder (IDD). The mean PRS for patients with and patients without IDD was 6.64E‐07 and 6.51E‐06, respectively (t = 0.361, P = 0.720). (D) PRS distribution plotted separately for patients with moderate, severe, and profound IDD; patients with mild IDD; and patients without IDD. The mean PRS for patients with moderate, severe, and profound IDD and those with mild IDD was 7.03E‐07 and 6.28E‐07, respectively (t = 1.78, P = 0.089). [file PCN-79-87-s003.docx]

**Supplementary**

**Whole-Genome Sequencing Analysis of Japanese Autism Spectrum Disorder Trios**

Running title: WGS of Japanese ASD Trios

**Supplementary Methods**

**Details of WGS data processing**

For data processing, we used ZettaVEGA, version 2.1.6.5772, on ZettaScaler 3.0 Server Unit hardware developed by PEZY Computing K.K. for variant calling. ZettaVEGA is a genome analysis pipeline that accelerates bwa-mem (https://github.com/lh3/bwa) and Genome Analysis Toolkit (GATK) Best Practice pipeline (https://gatk.broadinstitute.org/hc/en-us) software using a ZettaScaler 3.0 server unit equipped with a PEZY-SC3 processor. ZettaVEGA outputs are compatible with bwa-mem 0.7.17 and GATK 4.2.0.0. Alignment by pzBWA was performed with FASTQ, and alignment results were sorted using reshz sort, reshz MarkDuplicates, and reshz BQSR. pzHaplotypeCaller was executed with GATK-compatible parameters to obtain gVCF. Sequencing reads were aligned to Human Reference Genome hg38. For saliva samples, we used a bacterial decoy (MT169739.1) (1) to avoid calling sequences of non-human reads (oral bacteria). SNVs/INDELs were jointly called across all samples using the GenotypeGVCFs.

Variant call accuracy was evaluated using GATK VQSR. Depth was calculated using CollectWgsMetrics.

**Supplementary Tables**

Table S1. Input genes for enrichment analysis

Table S2. Clinical data for all patients with potentially pathogenic variants

Table S3. All SVs including CNVs overlapping exonic regions of ASD/IDD genes

**Supplementary Figures**

Figure S1. Ancestry confirmation

Multi-dimensional scaling (MDS) analysis was performed to exclude population outliers. PCA using the 1000 Genomes Project reference panel (phase 3) detected no subjects with probable ancestries outside the east Asian population and ensured that all probands were of apparent Japanese origin. Red, light green, pink, and yellow circles indicate CEU (Utah residents [CEPH] with Northern and Western European ancestry), CHB (Han Chinese in Bejing, China), JPT_1000G (Japanese in Tokyo, Japan), and YRI (Yoruba in Ibadan, Nigera) in 1000G, respectively. Blue circles indicate our sample.

Figure S2. Polygenic risk score (PRS)

A: PRS distribution of our patients and a sample of Japanese from 1000G (JPT_1000G). The mean PRS for our patients and JPT_1000G was 6.57E-07 and 6.51E-07, respectively (t = 0.26, *p* = 0.795). B: PRS distribution plotted separately for patients with and without potentially pathogenic variants. The mean PRS for patients with/without potentially pathogenic variants was 6.58E-07 and 6.56E-07, respectively (t = 0.037, *p* = 0.970). C: PRS distribution plotted separately for patients with and without IDD. The mean PRS for patients with and patients without IDD was 6.64E-07 and 6.51E-06, respectively (t = 0.361, *p* = 0.720). D: PRS distribution plotted separately for patients with moderate, severe, and profound IDD, patients with mild IDD, and patients without IDD. The mean PRS for patients with moderate, severe, profound IDD and patients with mild IDD was 7.03E-07 and 6.28E-07, respectively (t = 1.78, *p* = 0.089).

**Reference**

1. Samson CA, Whitford W, Snell RG, Jacobsen JC, Lehnert K. Contaminating DNA in human saliva alters the detection of variants from whole genome sequencing. Sci Rep. 2020;10(1):19255.
